# Deep learning radiomics based on contrast-enhanced ultrasound images for assisted diagnosis of pancreatic ductal adenocarcinoma and chronic pancreatitis

**DOI:** 10.1186/s12916-022-02258-8

**Published:** 2022-03-02

**Authors:** Tong Tong, Jionghui Gu, Dong Xu, Ling Song, Qiyu Zhao, Fang Cheng, Zhiqiang Yuan, Shuyuan Tian, Xin Yang, Jie Tian, Kun Wang, Tian’an Jiang

**Affiliations:** 1 CAS Key Laboratory of Molecular Imaging, The State Key Laboratory of Management and Control for Complex Systems, Institute of Automation, Chinese Academy of Sciences, Beijing, 100190 China; 2 School of Artificial Intelligence, University of Chinese Academy of Sciences, Beijing, 100049 China; 3 Department of Ultrasound, The First Affiliated Hospital, College of Medicine, Zhejiang University, Hangzhou, 310003 China; 4 The Cancer Hospital of the University of Chinese Academy of Sciences (Zhejiang Cancer Hospital), No.1 East Banshan Road, Gongshu District, Hangzhou, 310022 China; 5 Department of ultrasound, West China Hospital, Sichuan University, Chengdu, 610041 China; 6 Department of Ultrasound, Tongde Hospital of Zhejiang Province, Hangzhou, 310012 China; 7 Beijing Advanced Innovation Center for Big Data-Based Precision Medicine, School of Medicine and Engineering, Beihang University, Beijing, 100191 China; 8 Zhejiang Provincial Key Laboratory of Pulsed Electric Field Technology for Medical Transformation, Hangzhou, 310003 China

**Keywords:** Deep learning, Artificial intelligence, Pancreatic ductal adenocarcinoma, Contrast-enhanced ultrasound, Chronic pancreatitis

## Abstract

**Background:**

Accurate and non-invasive diagnosis of pancreatic ductal adenocarcinoma (PDAC) and chronic pancreatitis (CP) can avoid unnecessary puncture and surgery. This study aimed to develop a deep learning radiomics (DLR) model based on contrast-enhanced ultrasound (CEUS) images to assist radiologists in identifying PDAC and CP.

**Methods:**

Patients with PDAC or CP were retrospectively enrolled from three hospitals. Detailed clinicopathological data were collected for each patient. Diagnoses were confirmed pathologically using biopsy or surgery in all patients. We developed an end-to-end DLR model for diagnosing PDAC and CP using CEUS images. To verify the clinical application value of the DLR model, two rounds of reader studies were performed.

**Results:**

A total of 558 patients with pancreatic lesions were enrolled and were split into the training cohort (*n*=351), internal validation cohort (*n*=109), and external validation cohorts 1 (*n*=50) and 2 (*n*=48). The DLR model achieved an area under curve (AUC) of 0.986 (95% CI 0.975–0.994), 0.978 (95% CI 0.950–0.996), 0.967 (95% CI 0.917–1.000), and 0.953 (95% CI 0.877–1.000) in the training, internal validation, and external validation cohorts 1 and 2, respectively. The sensitivity and specificity of the DLR model were higher than or comparable to the diagnoses of the five radiologists in the three validation cohorts. With the aid of the DLR model, the diagnostic sensitivity of all radiologists was further improved at the expense of a small or no decrease in specificity in the three validation cohorts.

**Conclusions:**

The findings of this study suggest that our DLR model can be used as an effective tool to assist radiologists in the diagnosis of PDAC and CP.

**Supplementary Information:**

The online version contains supplementary material available at 10.1186/s12916-022-02258-8.

## Background

According to Global Cancer Statistics 2020, pancreatic cancer is the seventh leading cause of cancer-related death, with a five-year survival rate of less than 10% [1, 2]. Approximately 85–95% of pancreatic cancer patients have pancreatic ductal adenocarcinoma (PDAC) [3, 4]. Previous studies have shown that pancreatic cancer occurs more frequently in European and North American countries. The etiology is mainly attributed to genetic and environmental factors, especially diet and lifestyle, as well as a combination of factors such as obesity combined with smoking and alcohol [5, 6]. The poor prognosis in pancreatic cancer is due to a late diagnosis or misdiagnosis resulting from an overlap of symptoms with other conditions, such as chronic pancreatitis (CP) [7–10].

Imaging methods used in PDAC diagnosis include ultrasound (US), multidetector computed tomography (MDCT), magnetic resonance imaging (MRI), and positron emission tomography-computed tomography (PET-CT). Among them, contrast-enhanced ultrasound (CEUS) is convenient, poses no risk of radiation, and provides excellent spatial and temporal resolution to display microcirculatory perfusion of the pancreatic mass with parenchyma [11–16]. Moreover, studies have shown that PDAC can be distinguished from CP by comparing the enhancement intensity of the lesion to the pancreatic parenchyma during the venous phase [17–19]. However, the diagnostic performance of CEUS is largely dependent on the experience of radiologists. Furthermore, subjective imaging features and persistent inter- and intra-observer variability remain challenging factors in the interpretation of CEUS images [20, 21]. At present, there are few human experts who can consistently diagnose pancreatic disorders based on CEUS.

Radiomics is a method that extracts high-throughput quantitative features from medical images, which primarily use two analytical strategies in artificial intelligence (AI), machine learning, and deep learning [22–27]. The feasibility of radiomics in the diagnosis of PDAC has been demonstrated using MRI, computed tomography (CT), and endoscopic ultrasonography (EUS) images. Deng et al. [28] proposed a multi-parameter MRI radiomics model based on 119 patients with the best area under the curve (AUC) of 0.902 in the validation cohort to distinguish PDAC from CP. Ren et al. [29] verified the ability of texture analysis on unenhanced CT to distinguish PDAC from CP with best accuracy of 0.821. Tonozuka et al. [30] analyzed EUS images of 139 patients to distinguish among PADC, CP, and normal pancreas; the proposed deep learning radiomics (DLR) model achieved AUC of 0.924 and 0.940 in the validation and test cohorts. Although these studies show that the radiomics model can achieve good performance in the identification of PDAC and CP, several common limitations remain unaddressed. First, machine learning-based radiomics studies require labor-intensive and time-consuming lesion delineation, which inevitably is influenced by inter-and intra-operator reproducibility, especially in US images with unclear boundary definition [23]. Second, these studies did not investigate the actual benefits of using radiomics in real diagnostic scenarios for radiologists. Third, the feasibility of radiomics using CEUS imaging in diagnosing PDAC remains unverified.

This study was designed considering these limitations and aimed to (1) develop a DLR model for the automatic and accurate diagnosis of PDAC and CP using CEUS images and (2) validate the applicability of the DLR model as an effective tool to assist radiologists in the diagnosis of PDAC and CP. Additionally, the effect of this DLR model in assisting radiologists in decision-making is measured to assess its real clinical benefits. A two-round reader study with five radiologists was conducted to compare the diagnostic performance between the model and radiologists. More importantly, the ability of the model in assisting different radiologists identify PDAC and CP was investigated, which demonstrated its potential usefulness in real clinical practices.

## Methods

### Patients

This retrospective multicenter study was conducted using data from three hospitals in China (Hospital 1: First Affiliated Hospital, Zhejiang University School of Medicine; Hospital 2: Cancer Hospital of the University of Chinese Academy of Sciences; Hospital 3: West China Hospital, Sichuan University). It was conducted in accordance with the Declaration of Helsinki and approved by the ethics committee of each participating hospital. The requirement for informed consent was waived owing to the retrospective study design. This study followed the Standards for Reporting of Diagnostic Accuracy (STARD) guidelines for diagnostic studies.

The inclusion criteria were (I) patients with pathologically confirmed CP (followed up for at least 6 months without progression to pancreatic cancer) or PDAC without distant metastasis, (II) patients whose CEUS examination was performed within three days before biopsy and surgery, and (III) availability of CEUS video or CEUS images. The exclusion criteria were (I) multiple lesions in the pancreas, (II) with a history of pancreatic surgery or chemotherapy, and (III) inadequate CEUS image quality. All histopathological findings were confirmed by pathologists with more than 10 years of experience in pancreatic pathology.

**Fig. 1:**
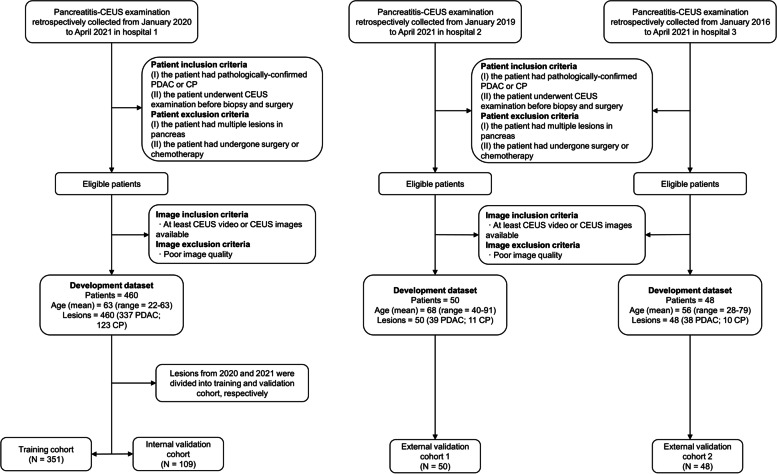
Retrospective workflow. CEUS, contrast-enhanced ultrasound; PDAC, pancreatic ductal adenocarcinoma; CP, chronic pancreatitis

Data derived from Hospital 1 with the largest number of enrolled patients were used as the primary cohort to reduce overfitting or bias in the analysis. In this study, patients of Hospital 1 were enrolled between January 2020 to April 2021. We selected the patients admitted in 2021 as the internal validation cohort and the patients admitted in 2020 as the training cohort. Data from Hospitals 2 and 3 were used as independent external validation cohorts. The detailed research process is illustrated in Fig. 1. Baseline characteristics including age, sex, lesion location and size, histological type, and carbohydrate antigen 19-9 (CA19-9) and carcinoembryonic antigen (CEA) levels were collected from the hospital database.

### Contrast-enhanced ultrasound image acquisition

Four different US devices (MyLab 90, ESAOTE, Italy; Aloka, HITACHI, Japan; LIGIQ E20, GE, USA; Resona 7, Mindray, China) equipped with an abdominal probe were used to capture the CEUS videos and/or images. Examinations were performed by one of the six radiologists with over 10 years of experience in abdominal CEUS. Before each examination, the proper contrast mode, including gain, depth, acoustic window, mechanical index, and focal zone, were adjusted. First, 2.4 mL of the contrast agent (SonoVue®; Bracco, Milan, Italy) was injected, followed by a 5-mL saline flush. The timer was started simultaneously when the contrast agent was being injected. Subsequently, the probe was kept in a stable state for 120 s to detect the pancreatic lesion and the surrounding pancreatic parenchyma. Finally, the video was recorded in Dicom format.

In this study, only one key CEUS image of each patient was finally selected for analysis. CEUS images of pancreatic lesions were mainly divided into three phases: vascular phase (0–30 s), pancreatic phase (31–60 s), and delayed phase (61–120 s) [14, 18]. Previous studies have shown that diagnosis of PDAC and CP using CEUS is mainly based on different enhancement patterns of the lesions. Studies have confirmed that during the pancreatic phase (30–40 s), the enhancement pattern could be high enhancement, equal enhancement, or low enhancement depending on the contrast of enhancement intensity between lesions and pancreatic parenchyma [13, 14, 31, 32]. Based on the above principles, we developed the criteria for the selection of key CEUS images. Owing to the retrospective nature of the study, dynamic CEUS video data of all patients were not completely preserved (half of the patients had no video). For maximal use of the existing data, image selection mainly included two schemes. For cases without dynamic video, 15–20 images were generally retained in the workstation during routine clinical work of CEUS examination in three participating hospitals, including important static CEUS images of three different phases. A typical static CEUS image of the pancreatic phase was selected for analysis, which showed the maximum diameter of the lesion at approximately the 35th second in duration. For cases with dynamic video, we directly selected the single frame around the 35th second in the dynamic video as a typical CEUS image for model development after preprocessing.

### Region of interest extraction and preprocessing

**Fig. 2:**
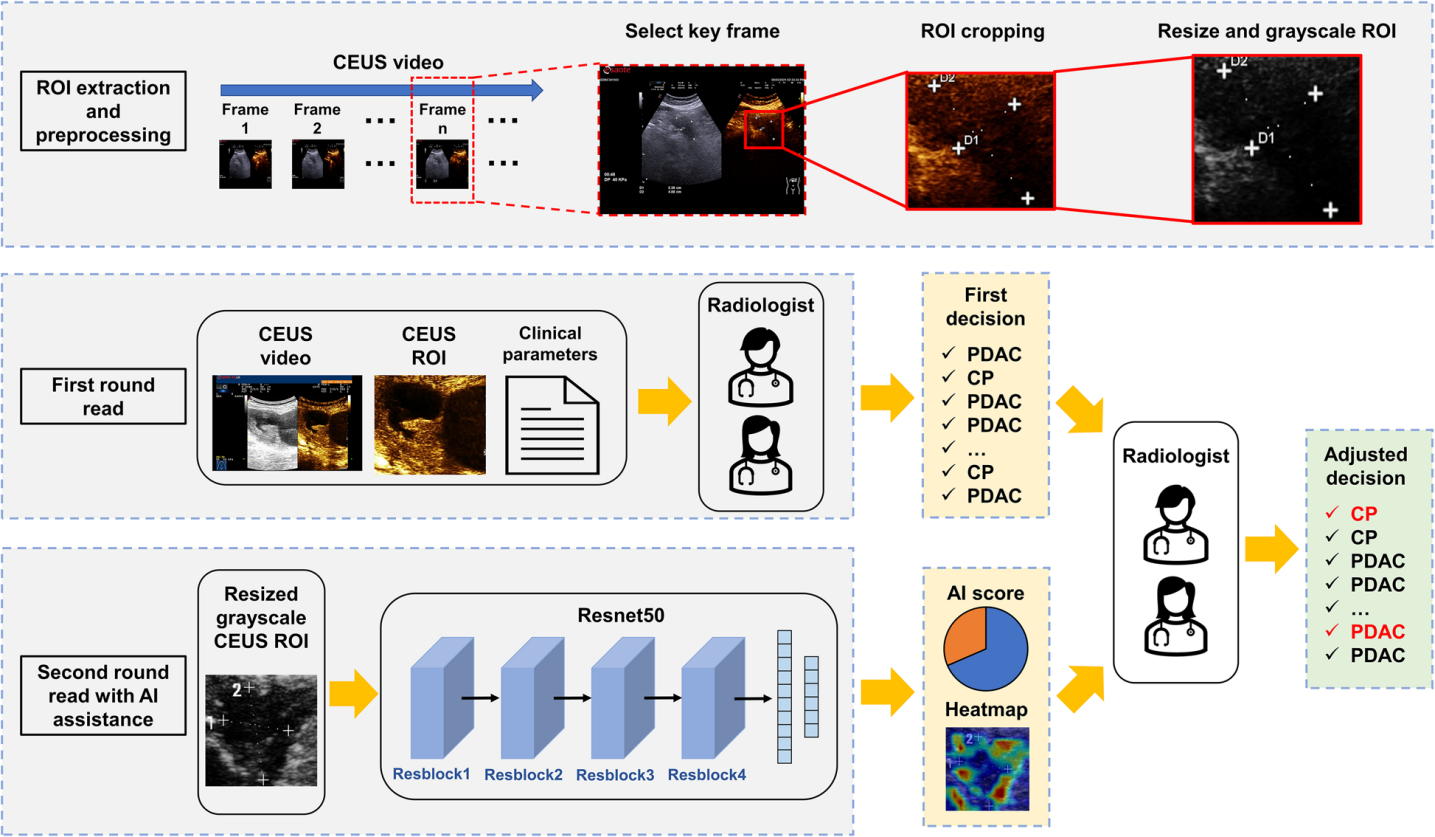
Workflow of ROI extraction and preprocessing and our DLR model. The ROI image is extracted from the raw CEUS video, if available; otherwise it is extracted directly from the existing CEUS images. The resized and grayscale ROI images are fed into our model which outputs the AI score and heatmap for each lesion. The radiologists provide an initial decision on each lesion and then adjust their decisions, if uncertain, based on the additional information provided by the DLR model. CEUS, contrast-enhanced ultrasound; PDAC, pancreatic ductal adenocarcinoma; CP, chronic pancreatitis; ROI, region of interest; AI, artificial intelligence; DLR, deep learning radiomics

The raw CEUS images were obtained by selecting the key frame from the CEUS videos or existing raw CEUS images extracted from the CEUS videos. Since two-dimensional (2D) grayscale US and CEUS images were displayed simultaneously in one view (Additional file 1: Fig. S1), we defined a rectangular region of interest (ROI) covering the lesion on the raw CEUS image, to eliminate the interference of irrelevant information from the image and non-lesion areas. The radiologist first determined the lesion area according to the 2D grayscale images in the raw CEUS images, following which the ROI was marked at the same location on the CEUS images. The open-source software labelme was used to label the ROI with a rectangular bounding box, and then the ROI image was cropped from the CEUS image [33]. In principle, the ROI image included the lesion and surrounding tissues. After the ROI extraction, further preprocessing was performed to obtain the resized and grayscale ROI images for model development. All colored ROI images were converted to greyscale, considering the color difference of the CEUS images collected from different US devices (Additional file 1: Fig. S2) and the minimal correlation between the enhancement pattern and color to improve the robustness of the DLR model for different equipment. Thus, only the distribution of the image gray values could affect the DLR model output. Finally, the grayscale ROI images were resized to 224×224 and inputted into the DLR model. The ROI extraction and preprocessing workflow is shown in Fig. 2.

### Deep learning radiomics model development

The DLR model was based on the Resnet-50 [34] backbone to extract deep learning features for classification (Fig. 2). Two fully connected layers with outputs of 512 and 2 neurons, respectively, and a softmax activation layer were placed on top of the convolutional layers to generate the AI scores for PDAC and CP. Using the softmax activation layer can give the AI score the meaning of probability, ensuring that the sum of the AI score in PDAC and CP categories for one lesion is 1. The dropout layer with a probability of 0.5 was added between every two fully connected layers to alleviate overfitting. Additional file 1: Table S1 illustrates the detailed architecture of our DLR model. We also tested other typical image classification backbones, including Inception-v3 [35], VGG-16 [36], and Densenet-121 [37]. The performance between different networks was very small in every cohort (Additional file 1: Fig. S3). Because Resnet-50 achieved the highest AUC in most validation cohorts, we chose Resnet-50 as the backbone for feature extraction. The detailed training process is provided in Additional file 1: Method S1 [38, 39].

### Two-round reader study

A two-round reader study was conducted to investigate the clinical benefits radiologists actually obtained through the assistance of the DLR model (Fig. 2). Five radiologists with an average of 9 years of CEUS experience (3–15 years) participated in this study. A total of 207 lesions (150 positives) from the internal validation cohort and the external validation cohorts 1 and 2 were presented in random order. During the whole process, the radiologists were blinded to each other, the original diagnostic reports, and the final pathology results. The details of the two-round reader study are provided in Additional file 1: Method S2 [40].

### Statistical analysis

Statistical analyses were performed using SPSS (version 23.0; IBM Corp., Armonk, NY, USA) and Python 3.7. Continuous variables were described as mean and standard deviation (SD), and categorical variables, as number and percentage. Between-group comparisons were performed using the Student’s *t*-test or Mann–Whitney *U* test for quantitative variables and the chi-squared test for qualitative variables. The 95% confidence interval (CI) was calculated using bootstrapping with 2000 resamples. The McNemar’s test was used to calculate whether the DLR model and the radiologists had significant differences in sensitivity and specificity. All statistical analyses were two-sided with statistical significance set at *P* <.05.

## Results

### Clinical data

In total, 558 patients with pancreatic lesions were enrolled (Fig. 1). Pathological findings showed PDAC lesions in 414 cases and CP lesions in 144 cases. Table 1 summarizes the detailed patient demographics and pancreatic lesion characteristics.

**Table 1:** Patient demographics and characteristics of pancreatic lesions

Characteristics	Training cohort (***N***=351)			Internal validation cohort (***N***=109)			External validation cohort 1 (***N***=50)			External validation cohort 2 (***N***=48)		
	PDAC (*n*=264)	CP (*n*=87)	*P*	PDAC (*n*=73)	CP (*n*=36)	*P*	PDAC (*n*=39)	CP (*n*=11)	*P*	PDAC (*n*=38)	CP (*n*=10)	*P*
Age (years, mean ± SD)	64 ± 9	62 ± 10	0.027	63 ± 11	63 ± 10	0.768	67 ± 11	66 ± 10	0.712	59 ± 12	48 ± 14	0.015
Sex			0.128			0.628			0.822			1
Male	161	45		39	21		25	6		26	7	
Female	103	42		34	15		14	5		12	3	
Location			0.248			0.117			0.543			0.379
Head or neck	139	52		35	23		15	6		27	5	
Body or tail	125	35		38	13		24	5		11	5	
Lesion size (mm, mean ± SD)	35.45 ± 12.98	37.34 ± 12.97	0.241	35 ± 23.04	32.25 ± 10.07	0.496	43.97 ± 11.70	35.72 ± 12.93	0.049	42.18 ± 15.94	33.4 ± 18.67	0.141
Laboratory data												
Serum CEA (ng/mL, median ± SD)	22.27 ± 88.54	10.49 ± 30.10	0.225	18.73 ± 57.04	41.02 ± 209.26	0.396	5.9 ± 5.78	3.75 ± 3.54	0.25	21.26 ± 84.46	6.44 ± 9.87	0.585
Serum CA19-9 (ng/mL, median ± SD)	3258.38 ± 4638.75	897.78 ± 2237.45	<0.001	2587.03 ± 4106.84	1379.50 ± 2927.84	0.118	2371.79 ± 8594.64	2587.75 ± 7858.74	0.941	1148.60 ± 4548.04	145.89 ± 321.94	0.493
Diagnosis			0.407			0.286			1			0.172
Surgery	79	22		14	4		5	1		15	3	
US-FNAB	185	65		59	32		34	10		23	7	

*PDAC* pancreatic ductal adenocarcinoma, *CP* chronic pancreatitis, *CEA* carcinoembryonic antigen, *CA19-9* carbohydrate antigen 199, *UC-FNAB* ultrasound-guided fine needle aspiration biopsy, *SD* standard deviation

### Comparison between deep learning radiomics model and radiologists

**Fig. 3:**
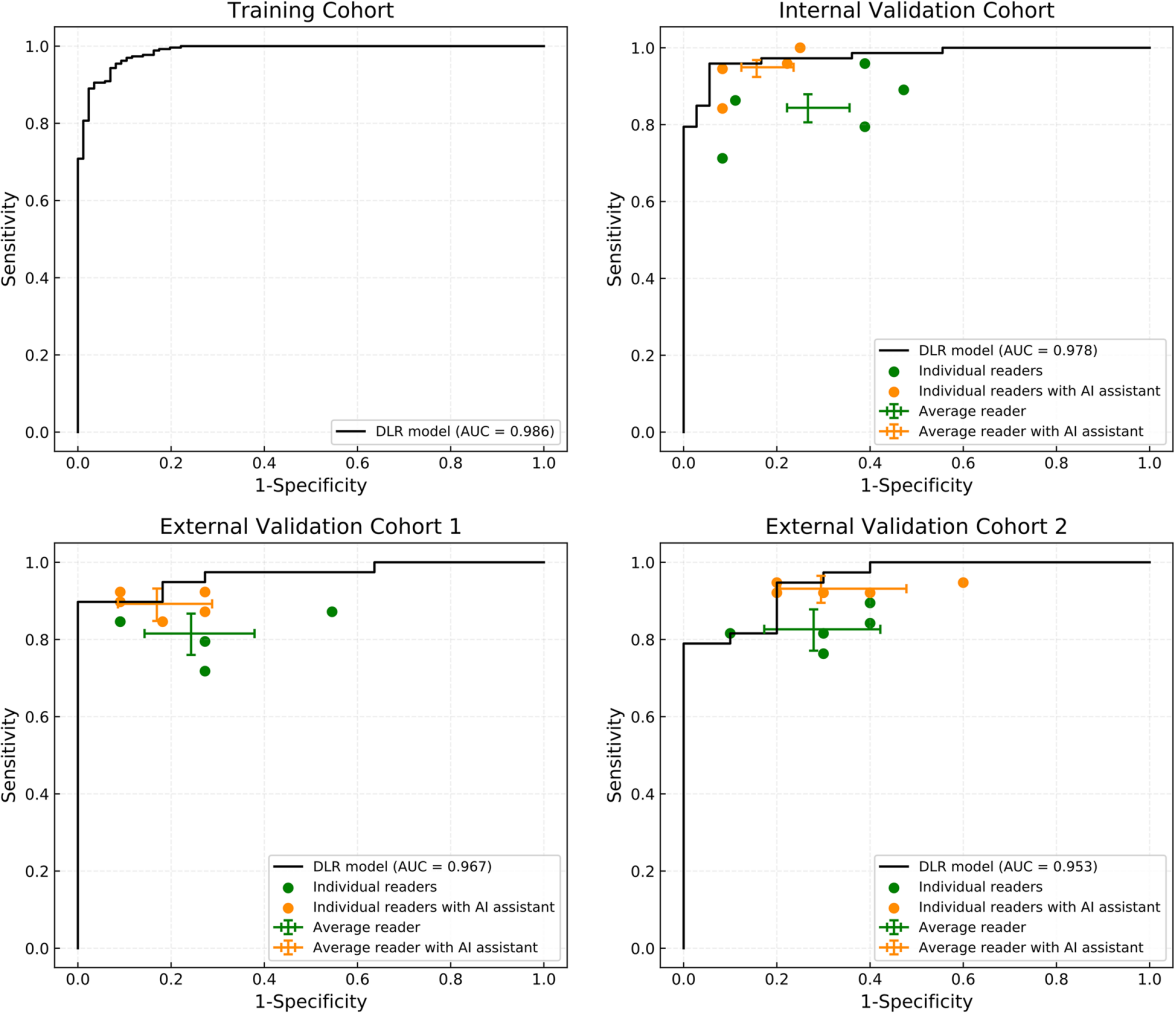
Comparison between performance of the DLR model and radiologists. The figure shows the identification of PDAC and CP in the training cohort, internal validation cohort, and external validation cohorts 1 and 2 using the DLR model and by individual radiologists. The performance of our DLR model is compared with each of the five readers and the average reader. DLR, deep learning radiomics; AUC, area under the curve; PDAC, pancreatic ductal adenocarcinoma; CP, chronic pancreatitis

The radiologists’ decisions from the first-round reading were compared with the DLR model. The receiver operator characteristic (ROC) curve of the DLR model, the diagnoses of each radiologist, and the average diagnostic results of all radiologists of the different cohorts are shown in Fig. 3. Our DLR model achieved a high AUC of 0.986 (95% CI 0.975–0.994), 0.978 (95% CI 0.950–0.996), 0.967 (95% CI 0.917–1.000), and 0.953 (95% CI 0.877–1.000) in the training, internal validation, and external validation cohorts 1 and 2, respectively. The sensitivity of internal validation, external validation cohort 1, and external validation cohort 2 were 97.3% (95% CI 93.2%–100%), 87.2% (95% CI 76.3%–97.2%), and 0.974 (95% CI 0.914–1.000); and the specificity values were 83.3% (95% CI 70.0%–94.3%), 100% (95% CI 100%–100%), and 70.0% (95% CI 37.5%–100%), respectively. The sensitivity and specificity results were based on the operation point of 0.5 [41]. The confusion matrices of DLR model are presented in Additional file 1: Fig. S4. Diagnoses of the five radiologists were either worse or comparable to those of the model. This is demonstrated by almost no green point reaching the upper left region of the ROC curve. Furthermore, average of all three reader diagnoses in the validation cohorts were located below the ROC curve of the model (Fig. 3, green crosses), revealing that our model was superior to the radiologists in general. The confusion matrices of the comprehensive diagnoses from the five readers without DLR assistance are presented in Additional file 1: Fig. S4.

For a more specific comparison, we also compared the sensitivity and specificity between the model and each radiologist. For fairness, we adjusted the operation point of the DLR model so that the specificity (sensitivity) matched the specificity (sensitivity) of each radiologist when comparing sensitivity (specificity). Since radiologists provide direct qualitative classification reports, sensitivity and specificity are fixed. The sensitivity and specificity of DLR model can be changed by adjusting the classification threshold. Based on the above principles, we achieved a specific comparison between the diagnostic performance of DLR model and radiologists. Detailed results are shown in Additional file 1: Table S2. In the internal validation cohort, the DLR model achieved better sensitivity and specificity than all radiologists, with a significantly higher sensitivity than three out of the five radiologists (*P* <.05 for Reader-1, Reader-2, and Reader-5) and a significantly higher specificity than three out of the five radiologists (*P* <.05 for Reader-2, Reader-3, and Reader-5). In the external validation cohort 1, the DLR model also achieved better sensitivity and specificity than all radiologists, with a significantly higher sensitivity than two out of the five radiologists (*P* <.05 for Reader-2 and Reader-5) and significantly higher specificity than Reader-1 (*P* <.05). In the external validation cohort 2, the DLR model achieved better sensitivity and specificity than all radiologists, except Reader-3. It showed a significantly higher sensitivity than two out of the five radiologists (*P* <.05 for Reader-2 and Reader-5), but not a significantly higher specificity.

### Enhanced diagnosis with AI assistance

The change in diagnoses given by the five radiologists before and after AI assistance were analyzed in the two-round reader study. Detailed changes in their decision, sensitivity, and specificity are shown in Table 2; and the confusion matrices of each radiologist without and with AI assistance are shown in the Additional file 1: Figs. S5 and S6. In the internal validation cohort, all radiologists achieved higher sensitivity, and four out of the five radiologists achieved higher specificity with AI assistance. Three and two of five radiologists had a significant improvement in sensitivity (*P* <.05 for Reader-1, Reader-2, and Reader-4) and specificity (*P*<.05 for Reader-2 and Reader-4), respectively. In external validation cohort 1, all radiologists achieved higher sensitivity, and two out of the five radiologists achieved higher specificity with AI assistance. In external validation cohort 2, all radiologists achieved higher sensitivity, and one out of the five radiologists achieved higher specificity with AI assistance. Reader-5 had a significantly higher sensitivity than the first-round results (*P*<.05). In all three validation cohorts, we found a positive effect of the DLR model in assisting radiologists to enhance their average accuracy (Fig. 3, orange points and crosses). Additionally, the confusion matrices of the comprehensive diagnoses of the five radiologists with AI assistance are given in the Additional file 1: Fig. S4.

**Table 2:** Summary of the changes in the decision-making of radiologists before and after AI assistance

	Reader number	True negative	True positive	CP ↓ PDAC^**a**^	PDAC ↓ CP^**b**^	Sensitivity (%)	Specificity (%)
Internal validation cohort	1	33→28	52→70↑	23	0	71.2→95.9↑*	91.7→77.8
	2	19→27↑	65→73↑	9	9	89.0→100.0↑*	52.8→75.0↑*
	3	22→27↑	70→73↑	3	5	95.9→100.0↑	61.1→75.0↑
	4	32→33↑	63→69↑	6	1	86.3→94.5↑*	88.9→91.7↑
	5	22→33↑	58→64↑	5	13	79.5→84.2↑	61.1→91.7↑*
External validation cohort 1	1	5→8↑	34→36↑	4	5	87.2→92.3↑	45.5→72.7↑
	2	8→8	31→34↑	4	1	79.5→87.2↑	72.7→72.7
	3	10→10	33→35↑	2	0	84.6→89.7↑	90.9→90.9
	4	10→10	33→36↑	3	0	84.6→92.3↑	90.9→90.9
	5	8→9↑	28→33↑	9	5	71.8→84.6↑	72.7→81.8↑
External validation cohort 2	1	7→7	31→35↑	6	2	81.6→92.1↑	70.0→70.0
	2	6→6	32→35↑	3	0	84.2→92.1↑	60.0→60.0
	3	9→8	31→35↑	7	2	81.6→92.1↑	90.0→80.0
	4	6→8↑	34→36↑	2	2	89.5→94.7↑	60.0→80.0↑
	5	7→4	29→36↑	13	3	76.3→94.7↑*	70.0→40.0

The upward arrow (↑) represents indicators that improved owing to AI assistance

*PDAC* pancreatic ductal adenocarcinoma, *CP* chronic pancreatitis, *AI* artificial intelligence

*P<.05

^a^Number of patients for whom the radiologists altered their decision from CP to PDAC

^b^Number of patients for whom the radiologists altered their decision from PDAC to CP

**Fig. 4:**
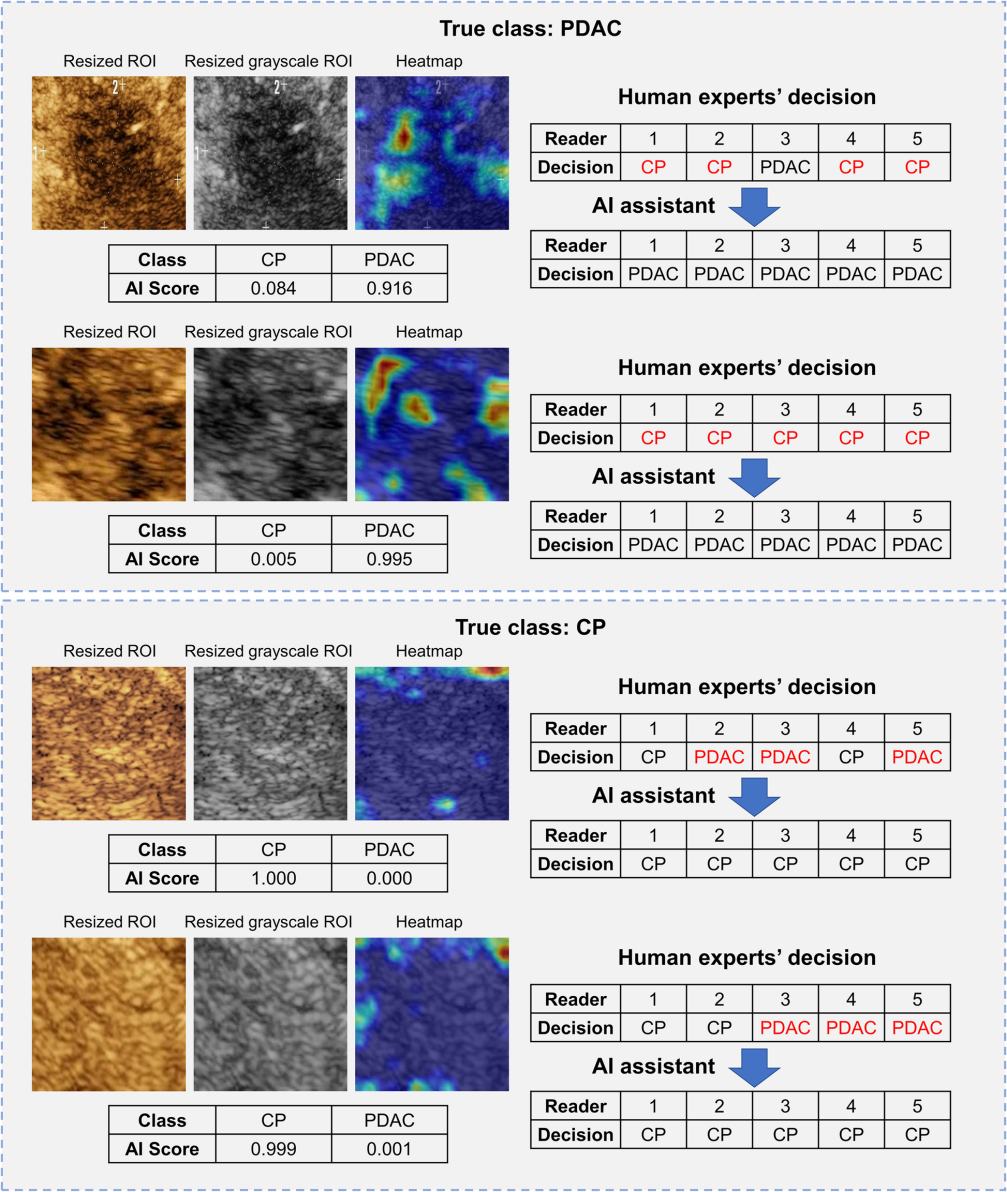
Typical cases of our DLR model guiding radiologists to make correct decisions. The top panel shows two PDAC lesions. Most radiologists consider these lesions as CP lesions in the first reading, but 100% accuracy is achieved with access to the additional information generated from the DLR model. In these two cases, the score of the DLR model for PDAC is significantly higher than that of CP, and the area of the highlighted regions is large in the heatmaps. Most of them are distributed inside the tumor, which is consistent with the regular pattern of the PDAC lesions found. The bottom panel shows two CP lesions. Most radiologists consider these lesions as PDAC lesions in the first reading, and 100% accuracy is achieved with access to the additional information generated from the DLR model. In these two cases, the DLR model scores CP significantly higher than PDAC, and the area of the highlighted regions is small in the heatmaps and mostly distributed at the boundary of the ROI image, which is consistent with the regular pattern of CP lesions found. PDAC, pancreatic ductal adenocarcinoma; CP, chronic pancreatitis; ROI: region of interest; AI, artificial intelligence; DLR, deep learning radiomics

**Fig. 5:**
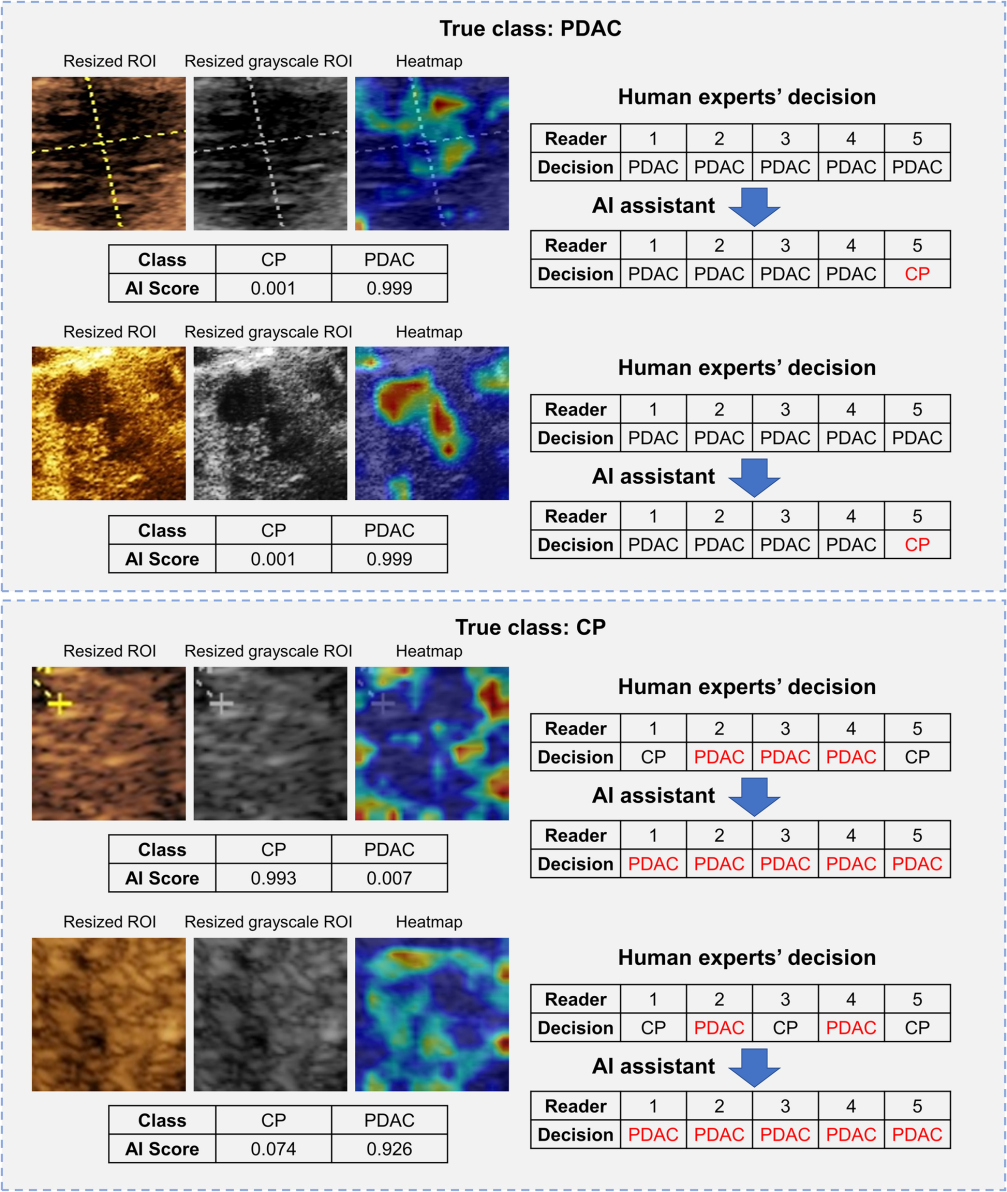
Typical cases of our DLR model that misled radiologists to make incorrect decisions. The top panel shows two PDAC lesions. All radiologists consider these two lesions to be PDAC lesions in the first reading. However, with access to the information from the DLR model, Reader-5 changed to the correct decision, considering them as CP lesions. In these two cases, the score of DLR model for PDAC is significantly higher than that of CP, and the area of the highlighted regions in the heatmaps are large and mostly distributed inside the tumor, which is consistent with other PDAC cases. Since Reader-5 is a junior radiologist, we believe that Reader-5’s mistakes may be due to lack of experience or carelessness. The bottom panel shows two CP lesions, which are inconsistent with the diagnosis of the radiologists in the first reading. However, with access to the information provided by the DLR model, all radiologists make the wrong decision. In these two cases, the misjudgment in the first case may be due to the large highlighted area of the generated heatmap, which is relatively rare in CP lesions, although most of the highlighted areas are still located at the boundary of the image. In the second case, the PDAC score with the DLR model is significantly higher than that of CP, which represents a case of AI misjudgment, thus misleading the radiologists. PDAC, pancreatic ductal adenocarcinoma; CP, chronic pancreatitis; ROI, region of interest; AI, artificial intelligence; DLR, deep learning radiomics

**Fig. 6:**
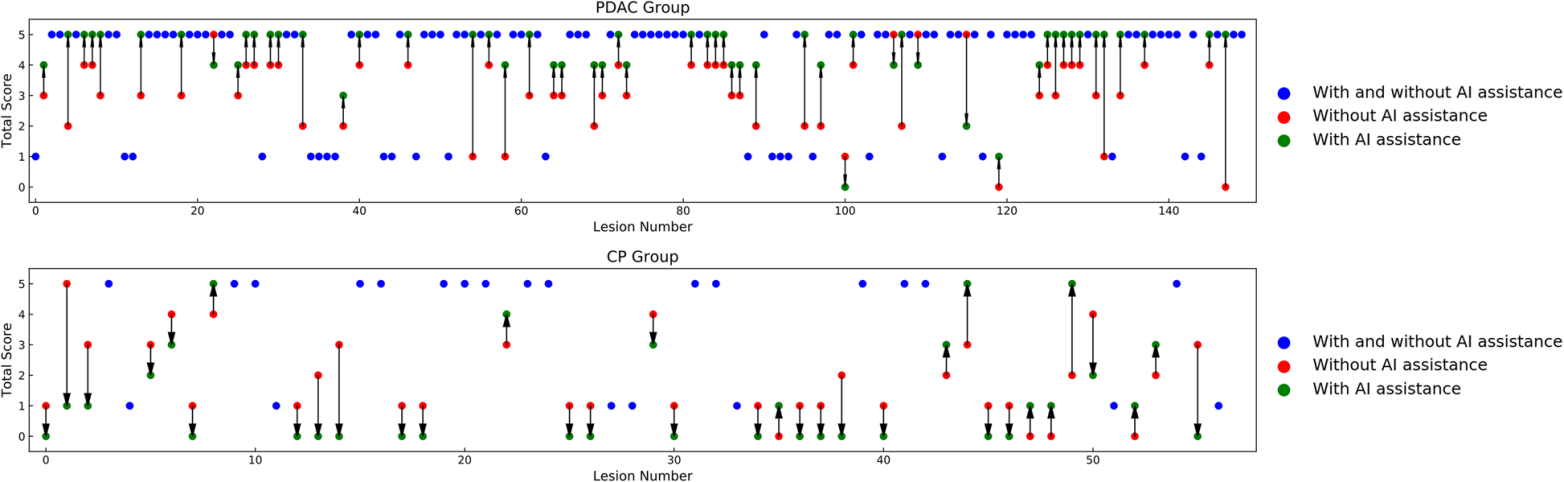
A summary of the total scores from five radiologists before and after DLR model assistance for every lesion in the validation cohorts. The red and green circles indicate the total score without and with DLR model assistance, respectively. The blue circles indicate that the lesion has the same score before and after AI assistance. The arrows indicate the trend of the total score after AI assistance. The total score is obtained by the sum of the scores of five radiologists individually. If a radiologist believes that a lesion is PDAC, it is scored as one point leading to a maximum score of five. The higher the score, the more experts believe that the lesion is PDAC. PDAC, pancreatic ductal adenocarcinoma; CP, chronic pancreatitis; AI, artificial intelligence; DLR, deep learning radiomics

To illustrate the clinical value of our DLR model more vividly, some successful and unsuccessful examples where radiologists changed their first-round decisions due to AI assistance are shown in Figs. 4 and 5. Although AI scores and heatmaps given by the DLR model misled the radiologists’ decisions in some cases, the total scores of the five radiologists for all lesions in the validation cohorts before and after DLR assistance exhibited a clear trend of enhanced diagnostic performance (Fig. 6). The total score was calculated as follows: if a patient was identified as a PDAC case by a radiologist, one point was awarded. Therefore, for five reads, the highest score was five, and the lowest score was zero. The higher the score, the more experts believed that the lesion was PDAC. The total scores demonstrated that a systematic improvement of the diagnostic accuracy was achieved in both PDAC and CP groups for all human experts with the help of the DLR model.

**Fig. 7:**
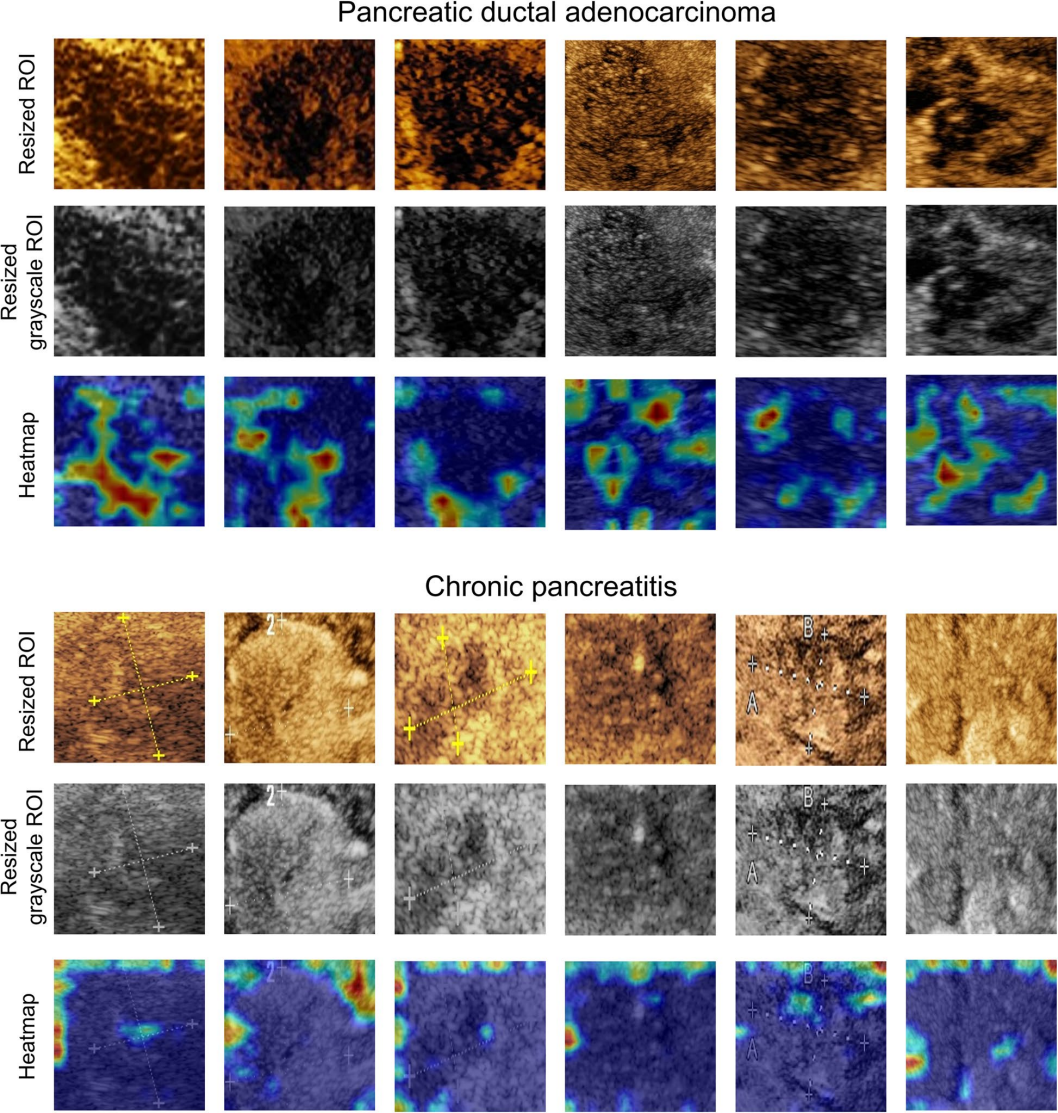
Examples of heatmaps generated by our DLR model for PDAC and CP lesions. Generally, the highlighted area of the PDAC lesions is larger than that of the CP lesions, and most of them are distributed inside the tumor. The highlighted areas are dominated by low-enhancement regions with adjacent high-enhancement regions around. The highlighted regions of the CP lesions are mainly distributed at the boundary of the image. This may be due to the lack of PDAC features in the center of the ROI. PDAC, pancreatic ductal adenocarcinoma; CP, chronic pancreatitis; ROI, region of interest; DLR, deep learning radiomics

Noticeably, the heatmaps generated by gradient-weighted class activation mapping for model visualization had different patterns in PDAC and CP images [40]. More specifically, the highlighted region for PDAC cases was greater than that of CP cases in the heatmaps, and most of those regions were located inside the lesions. In contrast, highlighted regions were mainly distributed at the boundary of the lesion in CP heatmaps. Additionally, radiologists noticed that for PDAC lesions, the highlighted regions were mainly distributed in the low-enhancement area inside the tumor, frequently adjacent to a high-enhancement region. Some heatmap examples of ROI images for PDAC and CP are shown in Fig. 7.

## Discussion

In this study, we attempted for the first time to investigate the performance of CEUS-based DLR in the diagnosis of PDAC and CP. Compared with human experts, our model achieved an overall better performance in all validation cohorts. Furthermore, we demonstrated that by incorporating AI scores and heatmaps, radiologists improved their decision-making, revealing the clinical value of applying the DLR model in clinical practice. Compared with other radiomics studies, a major highlight here was the use of the two-round reader investigation with five radiologists based on multicenter data.

The performance of our DLR model based on the CEUS images was better than or comparable to that of different models using other modalities, including MDCT, MRI, PET-CT, and EUS [28–30, 42]. This could be due to two possible reasons. First, compared with machine learning methods used in most of these studies [28, 29], the DLR model can automatically learn the adaptive features based on a specific task (effective identification of PDAC and CP) and it is flexible. Second, the diagnostic value of CEUS for PDAC has been demonstrated in previous studies [13, 43–45], confirming that the enhancement pattern in the lesion area contributes to qualitative diagnosis. Thus, it may contribute more to quantitative diagnosis.

Our DLR model achieved significantly higher, higher, or comparable sensitivity and specificity compared with the five radiologists in our first-round reader study. Although radiologists can identify lesions based on enhancement patterns, PDAC and CP may be difficult to distinguish when they exhibit similar CEUS enhancement patterns, mainly due to the presence of abundant fibrous tissue within PDAC lesions or necrosis within CP lesions. The DLR model can further learn and use high-level abstract features that are unrecognizable to humans to identify PDAC and CP, thus surpassing the diagnostic performance of human experts [24, 46–48].

Furthermore, we explored the benefits that radiologists actually obtained from the DLR assistance in clinical practice. We believe this is particularly important because DLR models will play a supporting role in the foreseeable future. Although AI and radiomics models have their superiorities, human experts would still make the final decision. One major reason is that the interpretability of deep learning features is still in its infancy [49, 50], and the biological mechanism behind these radiomics features remain underexplored. However, this should not stop radiologists from utilizing radiomics methods to enhance their diagnosis. In our design, AI scores notified radiologists about patients with different diagnoses between them and quantitative computer analysis. Heatmaps offered extra information for guiding their attention to the highlighted areas in the CEUS images so that they re-evaluated images more efficiently to decide whether to re-evaluate their decision. With this assisting strategy in the second-round image reading, human experts showed an overall increase in sensitivity to PDAC assessment with little or no loss of specificity.

We can understand how they helped radiologists effectively by investigating AI scores and heatmaps more thoroughly. The AI score can be regarded as the predicted probability of PDAC and CP by the DLR model. As can be seen from the frequency distribution histogram in Additional file 1: Fig. S7, we found that our DLR model provided a large ratio of extreme AI scores (e.g., greater than 0.9 for PDAC and less than 0.1 for CP lesions). As shown in Figs. 4 and 5, when the model provides an extreme score and strongly suggested the lesion are PDAC or CP, the AI score itself served as a strong indicator signal to the radiologists. The small ratio of ambiguous AI scores certainly helped with this “alarm” effect. Furthermore, heatmaps generated by DLR model reflected different patterns in the PDAC and CP lesions. For PDAC lesions, the highlighted areas were more concentrated in the low-enhancement region adjacent to the high enhancement area within the tumor, likely because of the DLR model learning key features from low-enhancement patterns related with less microvascular density, abundant fibrous tissue, and large amounts of necrotic tissue [51–54]. For CP lesions, since the model did not find important features towards PDAC, the highlighted area was relatively small and mainly distributed at the boundary of the ROI [55–59]. Therefore, the “alarm” effect and interpretable heatmap patterns together assisted radiologists to achieve real diagnostic benefits effectively.

Another potential clinical value of the DLR model is that it may help junior radiologists more effectively. Although all radiologists obtained positive assistance from the model, Reader-5, the junior radiologist, benefited the most. Therefore, this approach holds the potential to steepen the learning curve of radiologists with less experience.

Our study had several limitations. First, although this was a multicenter study, the dataset was not large, especially for the external validation cohort. Second, owing to the retrospective nature of the study, we did not use CEUS videos, which probably weakened the performance of the DLR strategy [14, 18]. Nevertheless, the strong performance of our model was sufficient to show that the use of static CEUS images provided effective clinical assistance.

## Conclusion

A DLR model for the diagnosis of PDAC and CP was developed from a multicenter retrospective dataset based on CEUS images. Further, a two-round reader study demonstrated that the model was effective in assisting radiologists to improve diagnosis.

## Supplementary Information

**Additional file 1: Method S1.** Detailed training process of our DLR model. **Method S2.** Details of the two-round reader study**. Figure S1.** One example of the raw CEUS image generated from the US device**. Figure S2.** Resized color and grayscale CEUS ROI images extracted from raw CEUS images generated by different US devices. **Figure S3.** Performance of different deep learning backbones on training and validation cohorts**. Figure S4.** Confusion matrices for the comprehensive results from five readers with and without DLR assistance and the DLR model on internal and external validation cohorts. **Figure S5.** Confusion matrices for Reader 1~5 without DLR assistance on internal and external validation cohorts. **Figure S6.** Confusion matrices for Reader 1~5 with DLR assistance on internal and external validation cohorts. **Figure S7.** Histogram representing the PDAC score output from the DLR model on CP and PDAC lesions. **Table S1.** The detailed architecture of our DLR model. **Table S2.** Sensitivity and specificity comparison between the diagnoses from the DLR model and that of each reader in the validation cohorts.

## Data Availability

The datasets analyzed during the current study are not publicly available due to the metadata containing information that could compromise the patients but are available from the corresponding author on reasonable request.

## Abbreviations

PDAC: Pancreatic ductal adenocarcinoma
CP: Chronic pancreatitis
DLR: Deep learning radiomics
CEUS: Contrast-enhanced ultrasound
AUC: Area under the curve
US: Ultrasound
MDCT: Multidetector computed tomography
MRI: Magnetic resonance imaging
PET-CT: Positron emission tomography-computed tomography
CEUS: Contrast-enhanced ultrasound
CT: Computed tomography
AI: Artificial intelligence
STARD: Standards for Reporting of Diagnostic Accuracy
CA19-9: Carbohydrate antigen 19-9
CEA: Carcinoembryonic antigen
2D: Two-dimensional
SDs: Standard deviations
CI: Confidence interval
ROC: Receiver operator characteristic
ROI: Region of interest
