# Supplementary material for: Deep learning radiomics based on contrast-enhanced ultrasound images for assisted diagnosis of pancreatic ductal adenocarcinoma and chronic pancreatitis
Source: BMC Med. 2022 Mar 2;20:74. doi: 10.1186/s12916-022-02258-8 (PMC8889703; doi:10.1186/s12916-022-02258-8)
Supplement: Supplementary file 1 — Additional file 1: Method S1. Detailed training process of our DLR model. Method S2. Details of the two-round reader study. Figure S1. One example of the raw CEUS image generated from the US device. Figure S2. Resized color and grayscale CEUS ROI images extracted from raw CEUS images generated by different US devices. Figure S3. Performance of different deep learning backbones on training and validation cohorts. Figure S4. Confusion matrices for the comprehensive results from five readers with and without DLR assistance and the DLR model on internal and external validation cohorts. Figure S5. Confusion matrices for Reader 1~5 without DLR assistance on internal and external validation cohorts. Figure S6. Confusion matrices for Reader 1~5 with DLR assistance on internal and external validation cohorts. Figure S7. Histogram representing the PDAC score output from the DLR model on CP and PDAC lesions. Table S1. The detailed architecture of our DLR model. Table S2. Sensitivity and specificity comparison between the diagnoses from the DLR model and that of each reader in the validation cohorts. [file 12916_2022_2258_MOESM1_ESM.docx]

# Additional file 1

# Supplementary Material

## Table of Contents

## Methods

Method S1. Detailed training process of our DLR model

Method S2. Details of the two-round reader study

## Figures

Fig. S1. One example of the raw CEUS image generated from the US device

Fig. S2. Resized color and grayscale CEUS ROI images extracted from raw CEUS images generated by different US devices

Fig. S3. Performance of different deep learning backbones on training and validation cohorts

Fig. S4. Confusion matrices for the comprehensive diagnoses from the five readers with and without DLR assistance and the DLR model on internal and external validation cohorts

Fig. S5. Confusion matrices for Reader 1~5 without DLR assistance on internal and external validation cohorts

Fig. S6. Confusion matrices for Reader 1~5 with DLR assistance on internal and external validation cohorts

Fig. S7. Histogram representing the PDAC score output from the DLR model on CP and PDAC lesions

## Tables

Table S1. The detailed architecture of our DLR model

Table S2. Sensitivity and specificity comparison between the diagnoses from the DLR model and that of each reader in the validation cohorts

Method S1. Detailed training process of our DLR model

The training objective of our DLR model was to minimize cross-entropy loss. The DLR model was developed based on the CEUS images in the training cohort with initial pre-trained weights from the ImageNet dataset [38] via transfer learning. During model development, online data argumentation was used to expand the number of samples in the training cohort, including random cropping, random rotation, random flipping, random contrast, brightness, and random translation. The DLR model was optimized using the adaptive moment estimation (ADAM) optimizer [39] at an initial learning rate of 0.0001, which then decayed in the 50^th^ epoch with a decay factor of 0.1. The maximum epoch was set to 100, with a batch size of 64 in each step. To overcome overfitting, L2 regularization was used with a weight decay value of 0.01. The final model weights were selected based on the AUC performance of the internal validation cohort after each epoch. Our method was implemented in Pytorch 1.0 and was performed on a personal computer with an Intel Core i9-9900k CPU, 64 GB memory, and one NVIDIA TITAN V GPU for computing acceleration.

Method S2. Details of the two-round reader study

In this study, a two-round reader study was performed to verify the clinical value of our DLR model. In the first round, the radiologists were only provided with the patient’s PDAC-related clinical characteristics (such as the level of tumor marker and patient demographics), raw CEUS video or images, and color and resized grayscale ROI images to make an initial diagnosis. The reading results in this round were compared with that of the DLR model directly to demonstrate the advantages of the DLR model. In the second round, we asked radiologists to refine their first-round of diagnoses with the additional AI score and heatmap information. Heatmaps were generated using the gradient-weighted class activation mapping (Grad-CAM) method [40] from the feature maps of the third residual block in the DLR model. The heatmap highlights the important regions where the DLR model is used for scoring the PDAC. The diagnoses in this round were compared with the diagnoses of the first round to compare and explore the benefits of the DLR model to radiologists.


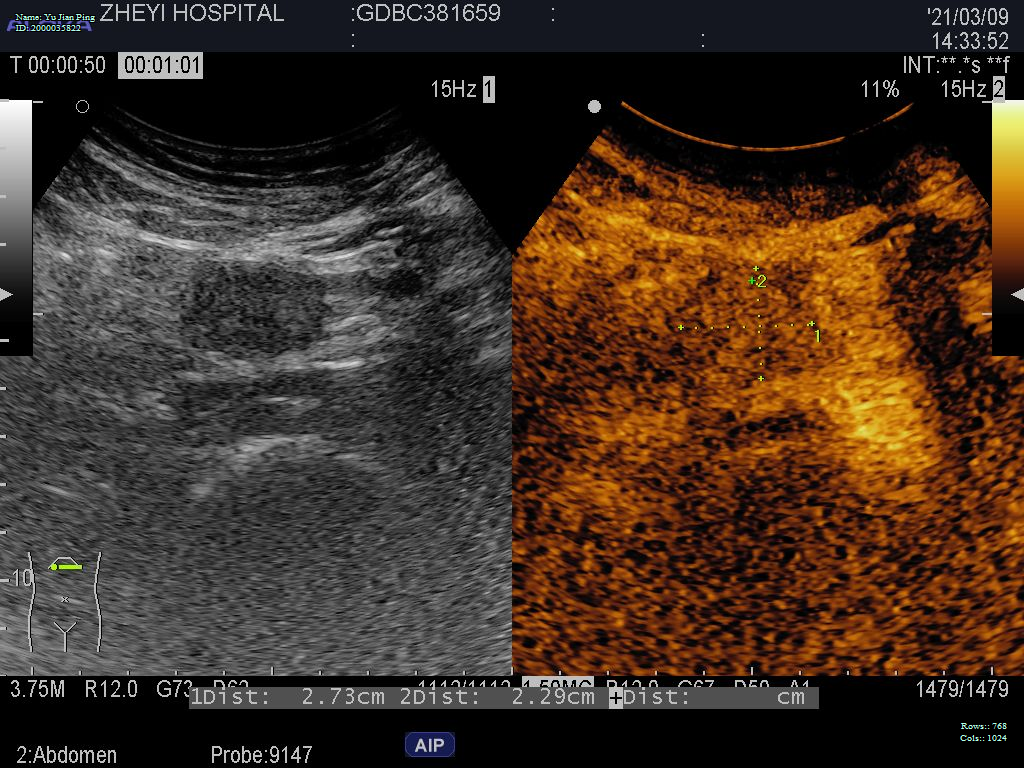


**Fig. S1. One example of the raw CEUS image generated from the US device**

The raw CEUS image contains two parts, namely the two-dimensional grayscale ultrasound image part and the CEUS image part.

CEUS: contrast-enhanced ultrasound; US: ultrasound


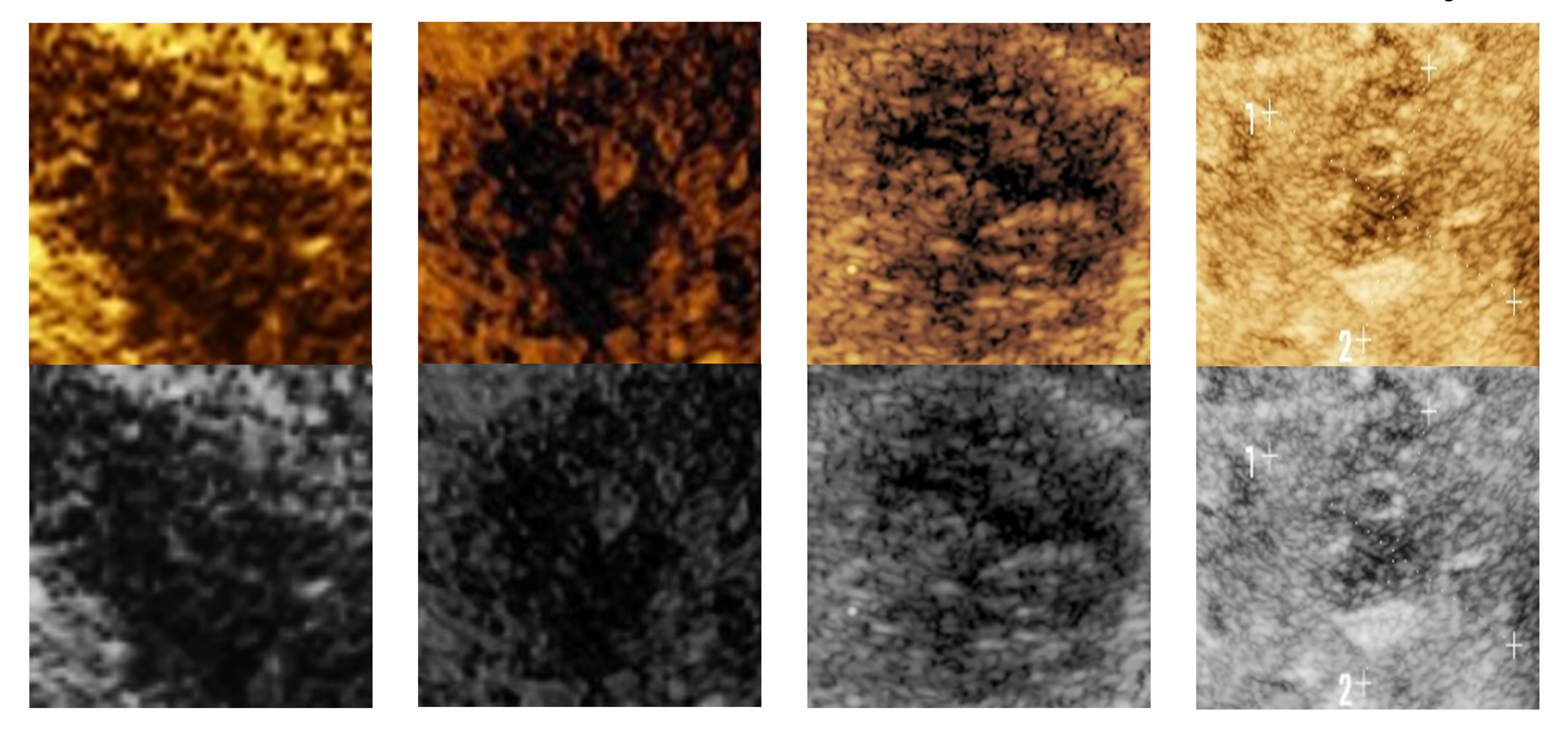


**Fig. S2. Resized color and grayscale CEUS ROI images extracted from raw CEUS images generated by different US devices**

The model and manufacturer of each US device from left to right is MyLab 90, ESAOTE, Italy; Aloka, HITACHI, Japan; LIGIQ E20, GE, American; Resona 7, Mindray, China.

CEUS: contrast-enhanced ultrasound; ROI: region of interest; US: ultrasound


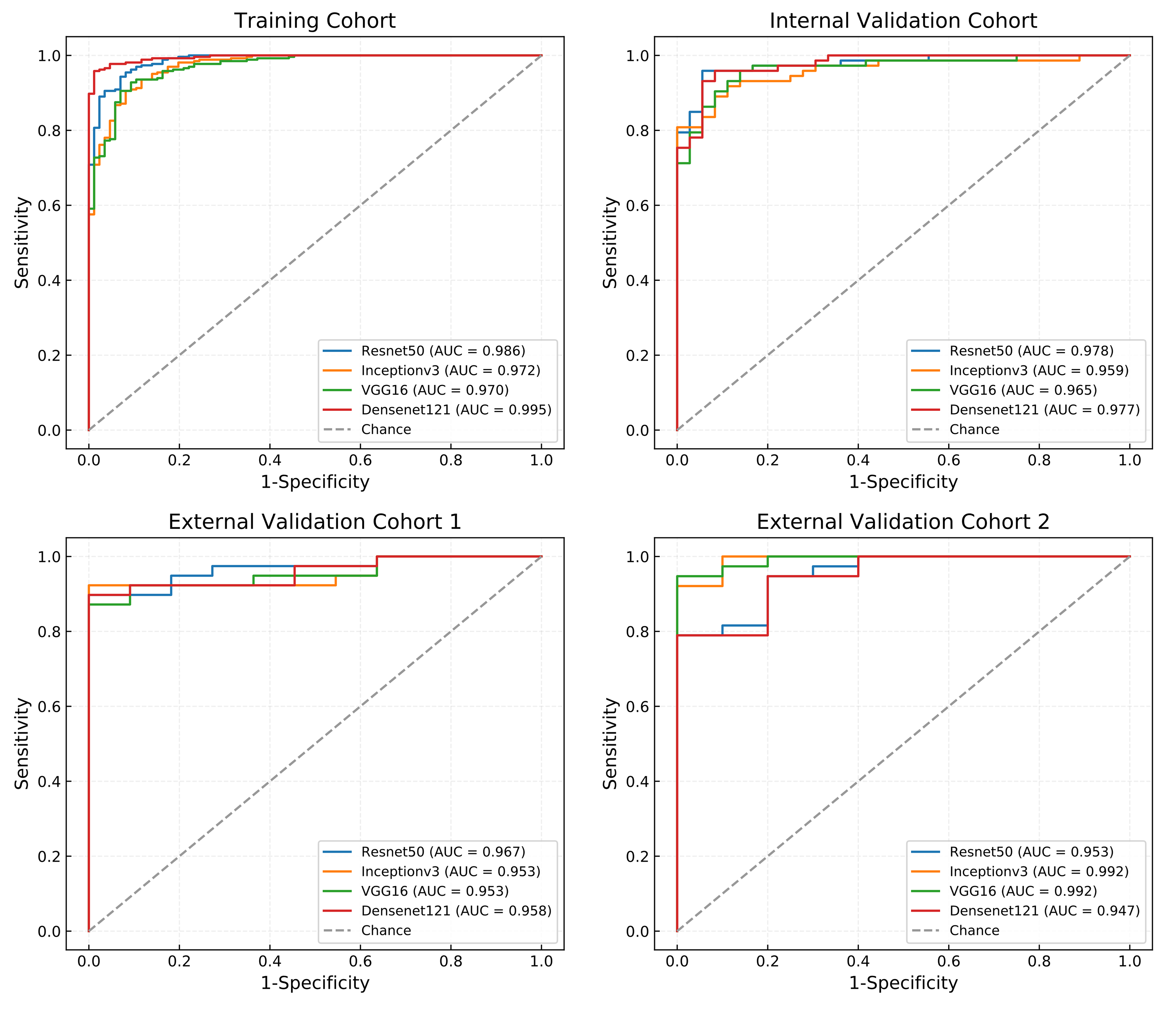


**Fig. S3. Performance of different deep learning backbones on training and validation cohorts**

All these models achieved a comparable diagnoses in every cohort. Since the Resnet-50 achieves the best AUC in most of the validation cohorts (internal validation cohort and external validation cohort 1), Resnet-50 was used as the backbone for feature extraction.

AUC: area under curve


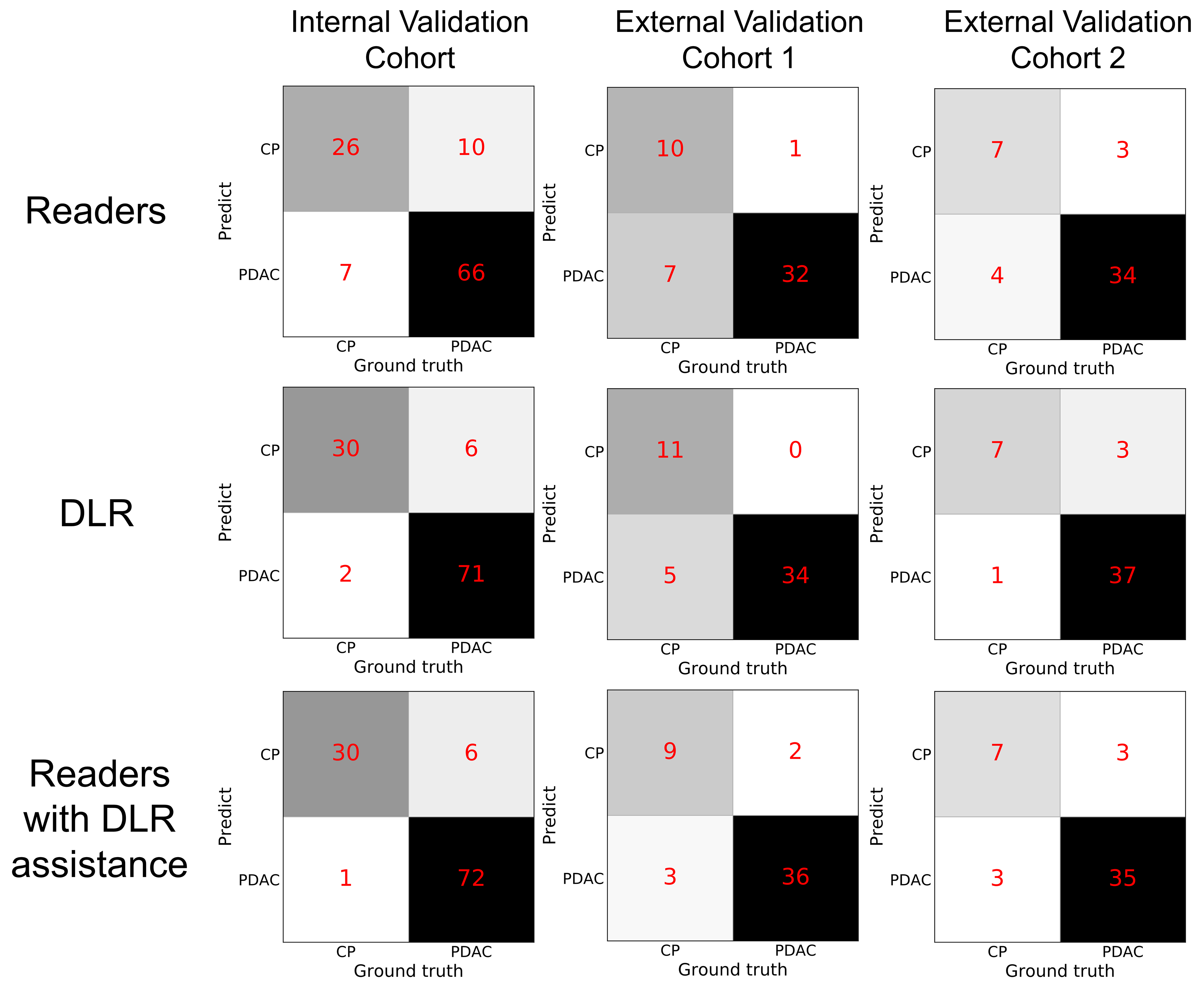


**Fig. S4. Confusion matrices for the comprehensive diagnoses from the five readers with and without DLR assistance and the DLR model on internal and external validation cohorts**

The comprehensive diagnoses from five readers were obtained based on their voting, that is, if there are three or more readers who agreed that the patient had PDAC, it was classified as PDAC, and if there are three or more readers who agreed that the patient had CP, then it was classified as CP. The confusion matrices generated from DLR model are based on the classification threshold of 0.5.

PDAC: pancreatic ductal adenocarcinoma; CP: chronic pancreatitis; DLR: deep learning radiomics


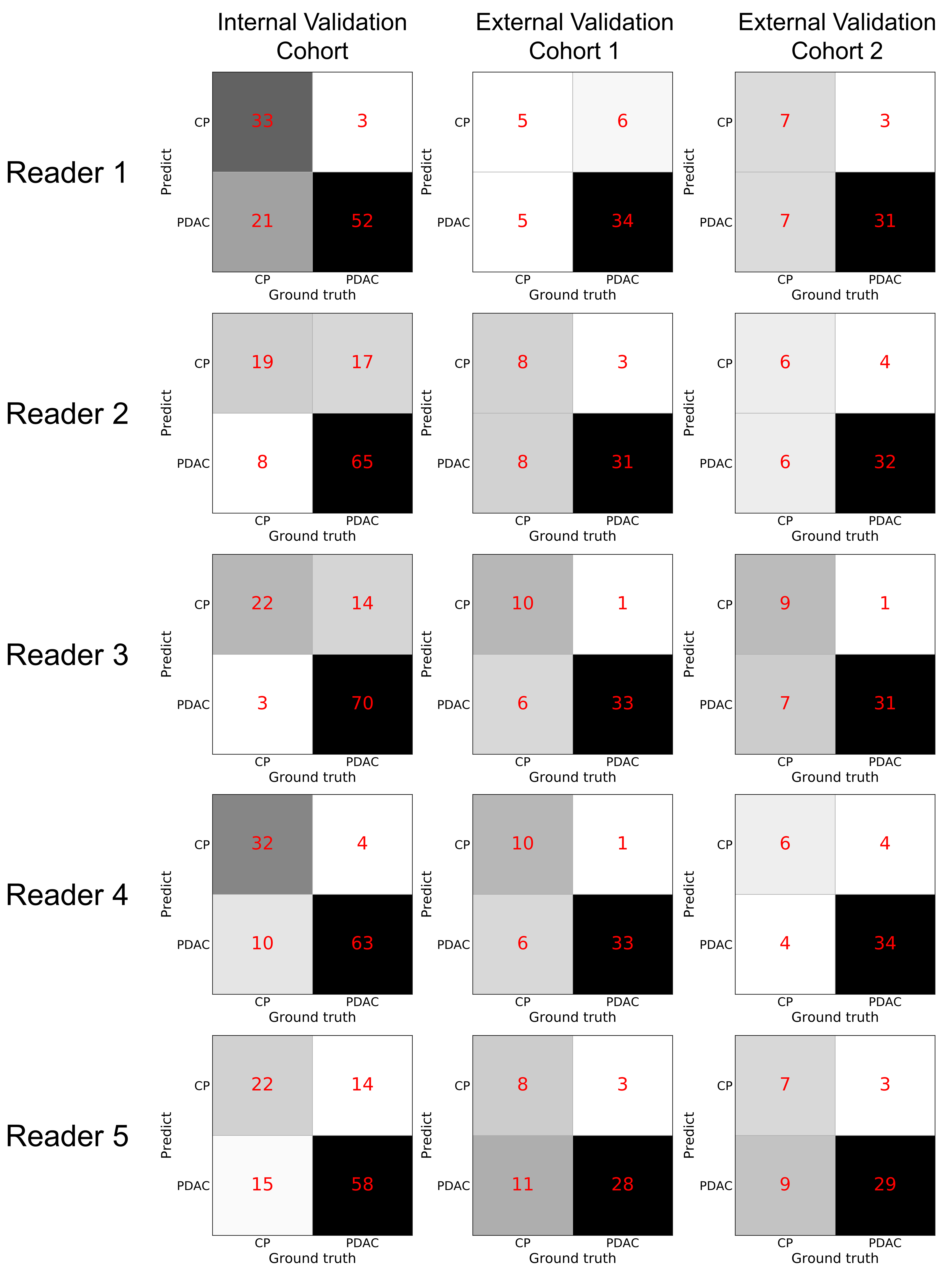


**Fig. S5. Confusion matrices for Reader 1~5 without DLR assistance on internal and external validation cohorts.**

PDAC: pancreatic ductal adenocarcinoma; CP: chronic pancreatitis; DLR: deep learning radiomics


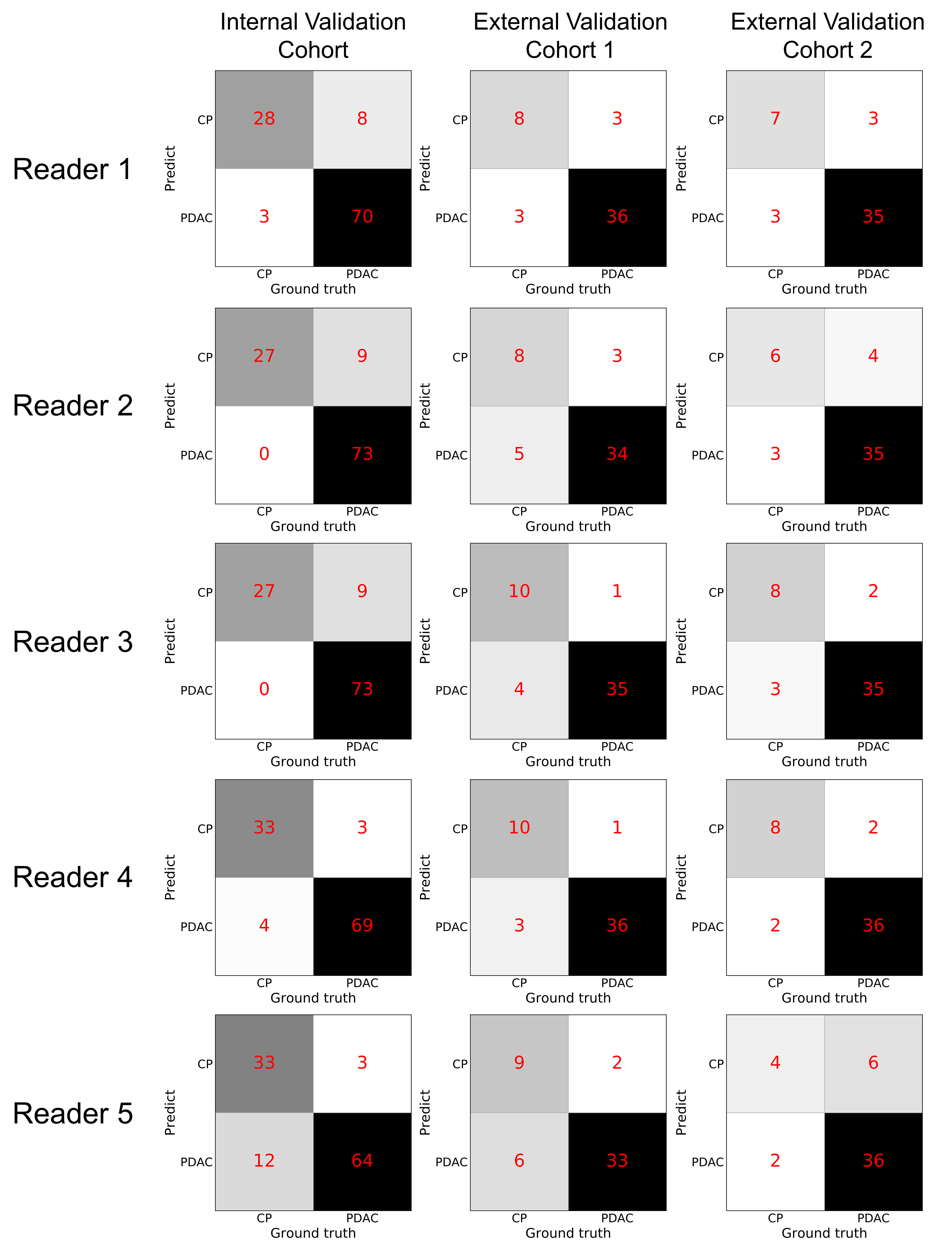


**Fig. S6. Confusion matrices for Reader 1~5 with DLR assistance on internal and external validation cohorts**

PDAC: pancreatic ductal adenocarcinoma; CP: chronic pancreatitis; DLR: deep learning radiomics


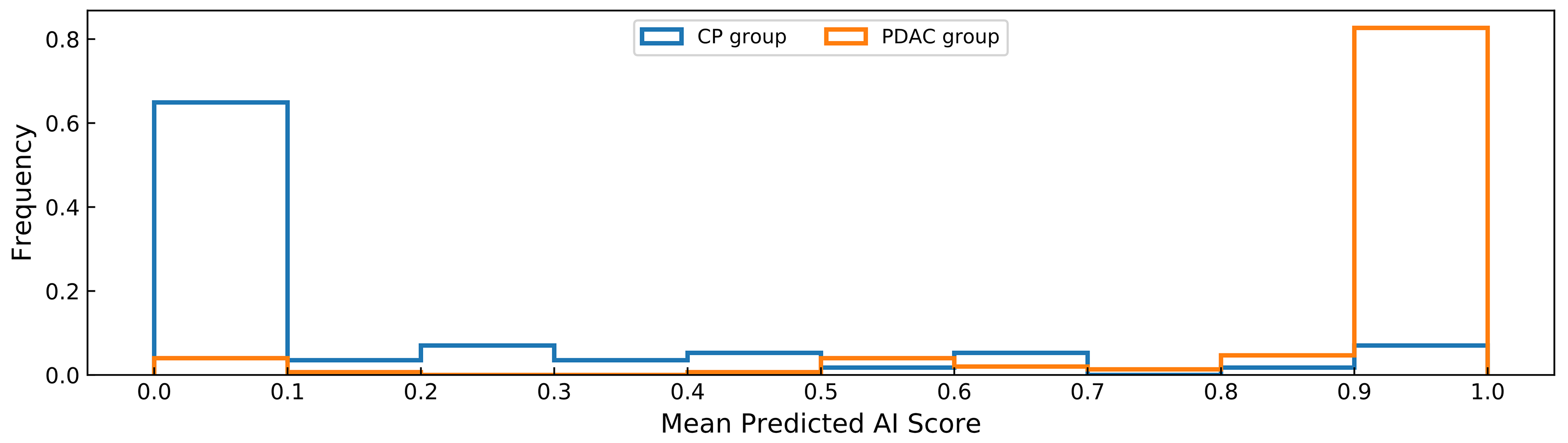


**Fig. S7. Histogram representing the PDAC score output from the DLR model on CP and PDAC lesions**

The AI scores contain a large ratio of extreme scores (e.g., greater than 0.9 for PDAC lesions and less than 0.1 for CP lesions). These extreme scores can be used as powerful signals to guide radiologists to make decision changes. At the same time, it should be noted that in some cases, the DLR model produced incorrect extreme probability, which may mislead radiologists. However, since the model itself has a high performance, the probability of this occurrence is very low.

PDAC: pancreatic ductal adenocarcinoma; CP: chronic pancreatitis; AI: artificial intelligence; DLR: deep learning radiomics

**Table S1. Detailed architecture of our DLR model**

| **Layer name** | **Output size** | **Description** |
| --- | --- | --- |
| Conv1 | 112 x 112 | 7 x 7, stride 2 |
| Maxpool1 | 56 x 56 | 3 x 3, stride 2 |
| Resblock1 | 56 x 56 | $\left[ \begin{matrix} 1\times1, 64 \\ 3\times3, 64 \\ 1\times1, 256 \end{matrix} \right]\times3$ |
| Resblock2 | 28 x 28 | $\left[ \begin{matrix} 1\times1, 128 \\ 3\times3, 128 \\ 1\times1, 512 \end{matrix} \right]\times4$ |
| Resblock3 | 14×14 | $\left[ \begin{matrix} 1\times1, 256 \\ 3\times3, 256 \\ 1\times1, 1024 \end{matrix} \right]\times6$ |
| Resblock4 | 7 x 7 | $\left[ \begin{matrix} 1\times1, 512 \\ 3\times3, 512 \\ 1\times1, 2048 \end{matrix} \right]\times3$ |
| GAP | 1 x 1 | 2048-d feature vector |
| Dropout1 | 1 x 1 | drop_rate = 0.5 |
| FC1 | 1 x 1 | 512-d feature vector |
| Dropout2 | 1 x 1 | drop_rate = 0.5 |
| FC2 | 1 x 1 | 2-d feature vector |

The layer written in red is different from the original Resnet-50 network.

DLR: deep learning radiomics, FC: fully connected

**Table S2. Sensitivity and specificity comparison between the diagnoses from the DLR model and that of each reader in the validation cohort**

|  |  | **Sensitivity (%)** | | | **Specificity (%)** | | |
| --- | --- | --- | --- | --- | --- | --- | --- |
|  | **Reader**  **number** | **Reader** | **DLR model**  **(95% CI)** | **P-value** | **Reader** | **DLR model**  **(95% CI)** | **P-value** |
| Internal  Validation  Cohort | 1 | 71.2 | 95.9  (89.9, 98.2) | **<0.0001** | 91.7 | 100.0  (100.0, 100.0) | 0.25 |
|  | 2 | 89.0 | 98.6  (95.7, 100.0) | **0.039** | 52.8 | 94.4  (86.0, 100.0) | **0.0002** |
|  | 3 | 95.9 | 98.6  (95.7, 100.0) | 0.625 | 61.1 | 94.4  (85.7, 100.0) | **0.002** |
|  | 4 | 86.3 | 95.9  (90.7, 100.0) | 0.065 | 88.9 | 94.4  (86.1, 100.0) | 0.688 |
|  | 5 | 79.5 | 98.6  (95.2, 100.0) | **0.0001** | 61.1 | 100.0  (100.0, 100.0) | **0.0001** |
| External  Validation  Cohort  1 | 1 | 87.2 | 97.4  (91.4, 100.0) | 0.125 | 45.5 | 100.0  (100.0, 100.0) | **0.031** |
|  | 2 | 79.5 | 97.4  (91.9, 100.0) | **0.016** | 72.7 | 100.0  (100.0, 100.0) | 0.25 |
|  | 3 | 84.6 | 89.7  (80.0, 97.6) | 0.625 | 90.9 | 100.0  (100.0, 100.0) | 1.0 |
|  | 4 | 84.6 | 89.7  (80.0, 97.6) | 0.625 | 90.9 | 100.0  (100.0, 100.0) | 1.0 |
|  | 5 | 71.8 | 97.4  (91.9, 100.0) | **0.002** | 72.7 | 100.0  (100.0, 100.0) | 0.25 |
| External  Validation  Cohort  2 | 1 | 81.6 | 97.4  (91.7, 100.0) | 0.07 | 70.0 | 90.0  (66.7, 100.0) | 0.5 |
|  | 2 | 84.2 | 100.0  (100.0, 100.0) | **0.031** | 60.0 | 80.0  (50.0, 100.0) | 0.5 |
|  | 3 | 81.6 | 81.6  (68.4, 92.9) | 1.0 | 90.0 | 90.0  (66.7, 100.0) | 1.0 |
|  | 4 | 89.5 | 100.0  (100.0, 100.0) | 0.125 | 60.0 | 80.0  (50.0, 100.0) | 0.5 |
|  | 5 | 76.3 | 97.4  (91.7, 100.0) | **0.021** | 70.0 | 100.0  (100.0, 100.0) | 0.25 |

To compare sensitivity, we adjust the operating point of the model based on the specificity of each reader so that the specificity of the model matches each reader. To compare specificity, we adjust the operating point of the model based on the sensitivity of each reader so that the sensitivity of the model matches each reader. The data in brackets are the 95% confidence intervals.

DLR: deep learning radiomics; CI: confidence interval
